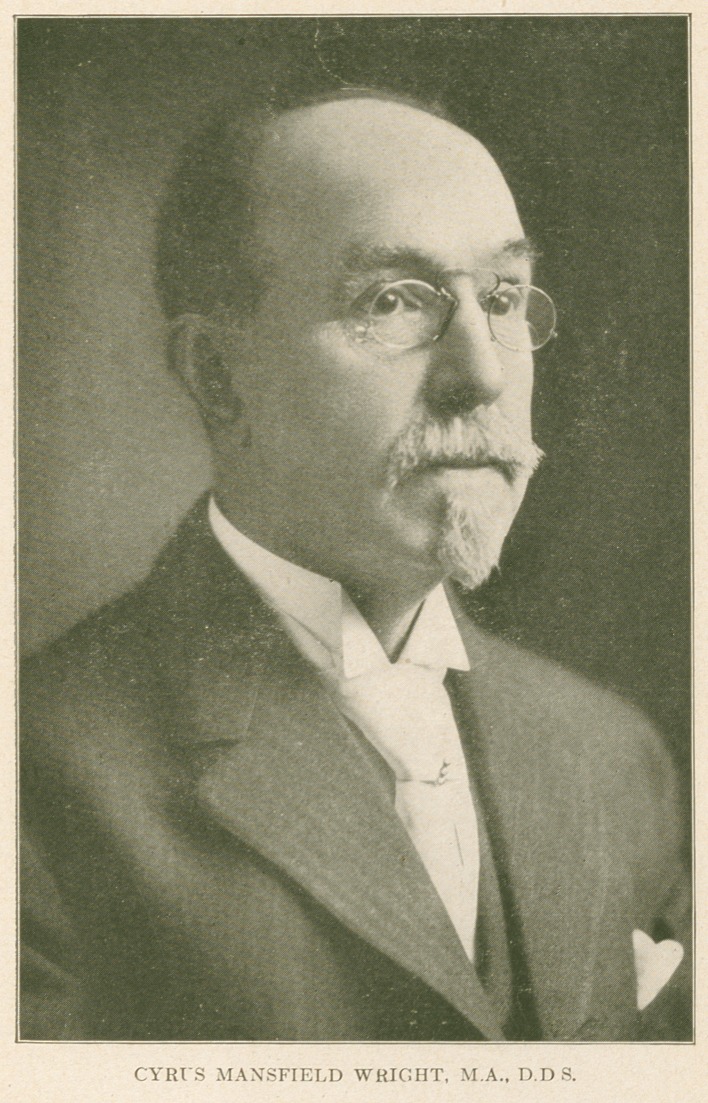# Cyrus Mansfield Wright, M.A., D.D.S.

**Published:** 1912-01-15

**Authors:** 


### CYRUS MANSFIELD WRIGHT, M.A., D.D.S.

Cyrus Mansfield Wright was born in Cincinnati, Ohio, on February 18, 1842. He attended the Chickering Preparatory School, and at the age of fifteen he became one of the Assistant Librarians of the Mercantile Library, where he read and studied with great avidity in all his leisure hours. Later he went to Oxford, Ohio, and was graduated in 1860 from Miami University, from which institution he also received the degree of M. A. in 1894.

In 1862 he was sergeant of Co. A, Brigade O. V. M. Two years later in 1864 he was graduated from the Ohio College of Dental Surgery in Cincinnati.

Having entered the office of Dr. George M. Keely in Oxford, Ohio, first as a student and then as a partner he remained with him until 1865 when he went to Cincinnati to become the partner of Dr. J. G. Cameron, with whom he remained until 1871, with the exception of a year or more spent with Dr. McClellan in Louisville experimenting with Rose Pearl.

Early in 1872 Dr. Wright bought and took over the practice of Dr. Van Marter in Basel, Switzerland. After fully establishing himself his active mind saw the necessity of closer associations among the American Dentists. He suggested to Drs. Van Marter, Williams, C. D. Terry and Geo. W. Field, the organization of a Society for their mutual benefit and for the sake of the value such a society would be to the profession. These men cordially agreed to the proposition, and on a bright day in July 1874 the little baby which has grown to such a vigorous manhood was born on the top of "The Rigi," and was christened, "The American Dental Society of Europe."

From the first the membership of this Society was limited strictly to men whose standing in the profession was of the highest rank and at present, with the same regard for professional ability and integrity it includes not only Americans from all parts of Europe, but prominent dentists of other nationalities.

The little Society developed slowly but steadily and when Dr. Wright was chosen its President at a meeting in Paris in 1877 he was greeted by a large and enthusiastic company of American Dentists.

CYRUS MANSFIELD WRIGHT, M.A., D.D S.

Preferring an American education and American environment for his children Dr. Wright returned in January of 1882, to take up his work again in Cincinnati, where he lived and practiced his profession until the day of his sudden death which was caused by a cerebral hemorrhage on November 15th, 1911.

Immediately upon his return to America he was elected to a Professorship in the Ohio College of Dental Surgery. The chair of Physiology and General Pathology he held continuously until he died, endearing himself to succeeding classes of young men and women.

During these almost thirty years he kept in close touch with the professional and literary life of his native city. He had held the office of President in both the "Mississippi Valley Dental Society" and the "Ohio State Dental Society" and was the author of several valuable works on Dentistry —one of these being, "Practical Hints about the Teeth"— which was widely printed in German and English. Dr. Wright was a constant contributor to Foreign and American Dental Journals and was always a welcome guest, reading papers of interest and importance at the annual meetings of the Illinois, Indiana, Kentucky and Ohio State Societies.

At the time of his death his term as President of the Cincinnati Literary Club had just expired. He held an honorary membership in the "United Chapters of Phi Beta Kappa Alumni."

Dr. Wright was a man of strong and pleasing personality vigorous and original and independent thinking, kind and generous, courteous to all. No words could more truthfully express the character of Dr. Cyrus Mansfield Wright than those fine words of Robert Louis Stephenson in his "Guidance of Life": "To be honest, to be kind; to earn a little and to spend a little less; to make upon the whole a family happier for his presence; to renounce, when that shall be necessary, and not to be embittered; to keep a few friends, but these without capitulation; above all, on the same given condition, to keep friends with himself,—here is a task for all that a man has, of fortitude and delicacy."

## Figures and Tables

**Figure f1:**